# Acknowledgement to Reviewers of the *International Journal of Environmental Research and Public Health* in 2014

**DOI:** 10.3390/ijerph120100455

**Published:** 2015-01-07

**Authors:** IJERPH Editorial Office

**Affiliations:** MDPI AG, Klybeckstrasse 64, CH-4057 Basel, Switzerland

The editors of the *International Journal of Environmental Research and Public Health* would like to express their sincere gratitude to the following reviewers for assessing manuscripts in 2014:

Ahluwalia, Indu
Ahmad, Farhan
Ahmed, Warish
Ajelli, Marco
Alavanja, Michael
Albadarin, Ahmad B.
Albrecht, Eric A.
Albrecht, Julie
Albrecht, Sandra S.
Alegria, Henry A.
Alegría, Margarita
Aletras, Vassilis
Alfonso, Helman
Alfred, Danita
Al-Gubory, Kaïs H.
Aller, Marta
Aller, Patricio
Ellenberger, F.
Almerich, Jose
Almquist, Ylva B.
Alonso-Magdalena, Paloma
Al-Sabi, Mohammad N.S.
lvarez, Debora
Alvarsson, Jesper
Amano, Yoshimasa
Amaral, Teresa F.
Amarasena, Najith
Amarenco, Pierre
Amato, Michael S.
Amengual Pou, Arnau
Amin, Raid
Ammenwerth, Elske
Anagnostopoulos, Fotios
Analitis, Antonis
Anangi, Balasubrmanyam
Ancona, Nicola
Andrade, Fernando
Andreotti, Gabriella
Andrews, Gavin
Andrews, Rob
Anenberg, Susan C.
Anido Rifón, Luis E.
Anton, Steve
Antonanzas, Fernando
Antonious, George F.
Antuna-Puente, Barbara
Antunes, Livia
Apekey, Tanefa
Apter, Alan
Arai, Masataka
Araujo, Rosa
Arcury, Thomas A.
Arduino, Matthew J.
Argos, Maria
Aris, Aziz
Armstrong, Ben
Arsene, Camelia
Arun, Nanditha
Asensi, Marise D.
Ash, Michael
Askew, Christopher D.
Athanasoulia, Anastasia
Augustin, Frankie
Ault, Steven
Austin, Elena
Autier, Philippe
Avery, Brent
Avogo, Winfred Aweyire
Ayele, Fasil Tekola
Baba, Sachiko
Backx, Frank J.G.
Badens, Catherine
Badera, Jarosław
Badieritakis, Evangelos G.
Bailey, Neil
Baker, Katherine
Baker, Laura
Bakke, Rune
Balbus, John
Balducci, Stefano
Baliatsas, Christos
Ballin, Scott
Bambrick, Hilary
Bammann, Karin
Banerjee, Areen
Banks, Jonathan
Barber, Bridget E.
Barbosa-Leiker, Celestina
Barcelos, Gustavo
Barker, Judith C.
Barlow, Karen Maria
Barnes, Jo M.
Barnes, Paul
Baronzio, Gianfranco
Barregård, Lars
Barrett, Paula
Barroso, João
Barthold, Julia
Barton, Jo
Bartram, Jamie
Basner, Mathias
Batchelor, Paul
Batista Rocha, Joao
Batista, Bruno Lemos
Battaglia, Luigi
Bauhoff, Sebastian
Baumgarth, Nicole
Baumgartner, Markus R.
Bayha, Keith
Beard, Emma
Beaver, John
Bednarska, Agnieszka J.
Beekhuizen, Johan
Beggel, Sebastian
Beil, Kurt
Belasco, Eric
Belinson, Jerome L.
Bell, Anita
Bell, Simon
Bellasio, Roberto
Belstrøm, Daniel
Belyaev, Igor
Bend, Jack
Benden, Mark
Ben-Dov, Iddo Z.
Benjamin, Stephanie
Benmarhnia, Tarik
Bennett, Kate M
Benninghoff, Abby D.
Benotsch, Eric
Benyamina, Amine
Bere, Elling
Bergin, Michael
Berkvens, Dirk
Bernard, Kristin
Bernd-Alois, Tenhagen
Berndt, Nadine
Bernier, Marie-Odile
Bernstein, Aaron
Berrigan, David
Berry, E. Helen
Berry, Peter
Bert, Fabrizio
Besse-Hoggan, Pascale
Betharia, Swati
Bethea, Traci N.
Bethel, Jeffrey
Beverland, Iain
Bharadwaj, Lalita
Bhargava, Pavan
Bhupathiraju, Shilpa
Bi, Peng
Bianchi, Fabrizio
Bielohuby, Maximilian
Bingham, Daniel
Bird, Nigel
Bisanzio, D.
Bischof, Gallus
Bischof, Werner
Biswas, Debabrata
Biziuk, Marek
Björk, J.
Björk, Jonas
Black, Mary Helen
Blanco, Juan Bautista
Blank, Michael
Bloch, Joan Rosen
Blum, Jason L.
Bobes, Julio
Bogdan, Christian
Bogstrand, Stig T.
Boisen, Nadia
Boisvert, Michelle
Bonasera, Stephen J.
Bonetta, Silvia
Boogaard, Peter
Borde, Theda
Borges, Aida
Borja-Aburto, Victor Hugo
Borm, Paul
Boschloo, Annemarie
Boschman, Julitta S.
Bot, Mariska
Bottoms, Lindsay
Boucher, Olivier
Bouwman, Lex
Bouzid, Maha
Box, Michael A.
Boyd, Carol
Boyd, Rhonda
Boyd, Windy
Boyer, Celia
Boyer, Laurent
Boyer, Treavor
Braack, Leo
Brandsema, Petra
Bråtveit, Magne
Braubach, Matthias
Brenner, Sara
Brent, Robert N.
Breysse, Jill
Bridges, Christy C.
Briner, Simon
Briner, Wayne
Bringas, Eugenio
Brink, Mark
Broadbent, Jonathan M.
Brody, Julia Green
Brokken, Leon
Brønnum-Hansen, Henrik
Bronsvoort, Mark
Brooks, Naomi
Brouwer, Rachel
Brown, David
Brown, Mary
Brown, Susan
Brucker, Natália
Bruckner, Tim A.
Brugge, Doug
Brumby, Susan A.
Brunak, Søren
Brüske, Irene
Brusseau, Timothy A.
Buckley, Timothy
Buckwalter, J. Galen
Bui, Hoan N.
Bultman, Scott J.
Bunch, Martin J.
Bungay, Kathleen
Buonanno, Giorgio
Burch, Tucker
Burgess, John A.
Burkart, Katrin
Burkhart, James F.
Burstyn, Igor
Burtscher, Martin
Butz, Arlene
Buunk-Werkhoven, Yvonne A. B.
Buxton, Jane
Byass, Peter
Byron, Justin
Bzik, David
Cabral, Horacio
Cai, Bo
Calderaro, Adriana
Calderón-Garcidueñas, Lilian
Calear, Alison
Calleja, Jesus M. Garcia
Calzo, Jerel P.
Camerino, Donatella
Campaile, Floriana
Campbell, David
Cancio, Ibon
Canetto, Silvia Sara
Cantonwine, David
Cao, Kai
Capelli, Gioia
Caplan, Susan
Capranica, Laura
Caputo, Fabio
Carlsen, Hanne Krage
Carmichael, Laurence
Carpenter, David
Carson, Kristin
Caruson, Kiki
Carvajal, M.
Casamassimo, Paul S.
Caserta, Donatella
Castillo-Rodal, Antonia Isabel
Castranova, Vincent
Cauchie, Henry-Michel
Cecchini, Michele
Cederbaum, Julie A.
Celico, Fulvio
Celo, Valbona
Ceschin, Simona
Chakraborty, Jayajit
Chalbot, Marie-Cecile G.
Chalifour, Lorraine
Chan, Catherine
Chan, P.W.
Chan, Ruby
Chang, Her-Kun
Chang, Howard
Chang, Hsien-Yen
Chang, Seo il
Chang, Ta-Yuan
Chang, Wei-chiao
Chang, Zheng
Chapman, Toni
Charles, Katrina
Charnock, Colin
Chatterton, Craig
Chau, K.W.
Chauhan, Preeti
Cheah, Nuan Ping
Chen, Binghen
Chen, Chin-fu
Chen, Han
Chen, Jie
Chen, Kow-Tong
Chen, Min
Chen, Szu-chieh
Chen, Wan-Qing
Chen, Xiaoli
Chen, Yuh-Fung
Chen, Zhuo
Chen, Zueng-sang
Cheng, June J.
Cheng, Po-Hsun
Cherry, Andrew L.
Cherry, Nicola
Chevreul, Karine
Chi, Kai Hsien
Chiang, Li-Chi
Chih, Ming-Yuan
Chih-Chien, Wang
Chillón, Palma
Ching, Wei-Mei
Chipps, Jennifer
Chirizzi, Daniela
Chiueh, P.T.
Cho, Sung-Hak
Choi, K.C.
Choi, Kelvin
Choi, Lillian
Choi, Yongrok
Choi, Young Sammy
Chow, Maria Yui Kwan
Chow, Winston T.L.
Chriqui, Jamie
Christensen, Hannah
Chu, Chun-Hung
Chu, Lawrence
Chuang, Cheryl C.Y.
Chuang, Kai-Jen
Cifra, Michal
Cimellaro, Gian Paolo
Cirino, Patrick
Claes, Neree
Claeson, Anna-Sara
Clark, Bronwyn
Clark, Robin
Clarke, Malcolm
Clements, C. John
Clougherty, Jane
Cochrane, Tom
Cohen, Deborah
Cole, Matthew
Coleman, B.L.
Coleman, David
Collado, Silvia
Collett, Brent R.
Collier, Abby
Collier, Scott R.
Collings, Paul
Collins, Pamela Y.
Collins, Timothy
Collins-Emerson, Julie
Comasco, Erika
Comelli, Elena M.
Conti, Susanna
Cook, Bryan G.
Cook, C. Justin
Cook, Elizabeth M.
Cook, Penny
Coombs, Geoffrey W.
Cooper, Rebecca
Corbelle Rico, Eduardo Jose
Corbett, Blythe A.
Cory-Slechta, Deborah A.
Cosnier, Frédéric
Costa, Silvia
Couchoud, Cécile
Covell, Nancy H.
Cox, Georgina
Craig, Eva
Crary, Michael
Creanga, Andreea A.
Creanza, Nicole
Creed, Torrey
Crimmins, Bernard
Crist, Richard
Cronk, Ryan
Cross, Amanda J.
Cservenka, Anita
Cuadros, Diego
Cuberek, Roman
Cui, Bianxiao
Cui, Jian
Culp, Ken
Cummings, K. Michael
Cummins, Robert A.
Cunningham, Frances C.
Currid, Thomas
Curtis, Andrew
Curwin, Brian
Cyrys, Josef
Dahdah, Nagib
Dai, Yun
Dalla Vecchia, Laura
D'Alonzo, Karen T.
Dalton, Alice M.
Dalvie, Mohamed Aqiel
Dancer, Stephanie
Daniel, Milan
Danieli, Pier Paolo
Dannenberg, Andrew
Darbre, P.D.
Darchen, André
Das, Ipsita
Dash, Aditya P.
Dassenakis, Μanos J.
Dauvrin, Marie
Davidson, Timothy M.
Davis, Elizabeth E.
Davis, Helen L.
Davis, Robert E.
Dawson, Kenneth A.
Day, Ashley
de Figueiredo, Daniela R.
de Franca Doria, Miguel
de Hoogh, Kees
De Jong, Elske
De La Rosa, Mario
de Lunaa, Mark Daniel G.
De Martelaer, Kristine
de Mesnard, Louis
De Sario, Manuela
de Souza, Lorraine H.
de Vocht, Frank
De Vogli, Roberto
De Zwaan, Martina
Decker, Brooke
Deininger, Prescott L.
Deka, Devajyoti
Dekkers, Susan
Delavari, Maryam
Delbosc, Alexa
Delmelle, Eric M.
Delva, Jorge
Demeke, Tigst
Dennekamp, Martine
Depcik, Christopher
Derman, Peter
Derose, Kathryn Pitkin
Desbois, Andrew
Desenclos, Jean-Claude
Desvars, Amel
Devís-Devís, José
Devlin, Robert
Dewan, Ashraf
Di Giulio, Mara
Diamanti-Kandarakis, Evanthia
Diaz, Esperanza
Diaz, G.J.
Diaz, Julio
DiBonaventura, Marco
Dickey, Bret M.
Dickinson, Miriam
Dickinson, Tommy
Díez-Manglano, Jesús
Dilley, Julia A.
Dino, Geri
Diraco, Giovanni
Dirks, Kim N.
Divaris, Kimon
Dobbler, Gerhard
Dohi, Yasuaki
Dollman, James
Dolnicar, Sara
Dombrowski, Kirk
Domínguez, Angela
Domschke, Katharina
Donald, Graeme
Donaldson, Alex
Donath, Lars
Dong, Huijuan
Dong, Jiang
Dong, Xuhui
Dong, Zhao
Donnell, Greg E.O.
Dons, Evi
Dorado Castano, Antonio David
Dorea, C.C.
Dorevtich, Sam
Dorri, Mojtaba
Dove, Melanie
Drake, Bettina F.
Dreibelbis, Robert
Drenowatz, Clemens
Driessen, Peter
Droge, Steven T.J.
Du, Hongfei
Du, Yifeng
Duan, Weili
Ducatman, Alan
Duer, Wolfgang
Duffy, Linda
Dumitrescu, Oana
Eagles, Paul F.J.
Eakin, Michelle N.
Eckel, Sandrah P.
Edward, Anbrasi
Edwards, Joshua R.
Eek, Frida
Efird, Jimmy
Egloff, Ann Marie
Eisenberg, Daniel
Ekenga, Christine C.
Ekerljung, Linda
Ekuni, Daisuke
El-Aty, A.M. Abd
Eleftheriou, Eleftherios
El-Geneidy, Ahmed
Elmenhorst, Eva-Maria
El-Sayed, Abdulrahman M.
Elvik, Rune
Emberger, Günter
Emeis, Stefan
English, Paul
Erdely, Aaron
Erickson, Marilyn
Escoffery, Cam
Etz, Kathleen E.
Evensen, Katinka
Ewa, Bok
Exley, Chris
Eyrolle-Boyer, Frédérique
Ezekowitz, Justin A.
Fabian, Patricia
Factor-Litvak, Pam
Falconer, Ian R.
Falkinham III, Joe O.
Fan, Yingling
Fanizza, Carla
Farber, Harold J.
Farr, Sherry
Farren, Conor K.
Farsalinos, Konstantinos
Fath, Brian
Fatin-Rouge, Nicolas
Fattore, Elena
Favretto, Donata
Fazzo, Lucia
Fedio, Willis M.
Felthous, Alan R.
Fenner, Kathrin
Ferketich, Amy
Fernández Parra, Antonio
Ferreira, Isabel
Figueras, Maria-José
Filion, Kristian B.
Filipiak, Krzysztof
Finch, Caleb E.
Fink, Rok
Finkel, Madelon
Finkelstein, Fedric
Finlayson, Brian
Finneran, Kevin
Fischer, Gloria
Fischer, Joachim E.
Fleig, Lena
Fleischauer, Aaron
Fleischhacker, Sheila
Fleming, Carrie
Floros, Georgios
Foley, Janet
Font, Laia
Foraker, Randi E.
Ford, Judy
Ford, Stephen
Formisano, Pietro
Fortoul, Teresa I.
Fourches, Denis
Fox, Bradley K.
Fox, Mary A.
Francey, Thierry
Francis, Robert A.
Franco, Rodrigo
Frank, Andrew
Frantzeskou, Elpida
Fraqueza, Maria J.
Frattaroli, Shannon
Freedman, David S.
Freeman, Matt
Frew, Paula
Friesen, Claudia
Friesen, Melissa C.
Friger, Michael
Friguls, Bibiana
Friswell, Rena
Fröhlich, Eleonore
Fry, Rebecca
Fry, Rebecca C.
Frye, Victoria
Fu, Dongbo
Fu, Qiang
Fu, Xiuju
Fuermaie, Anselm B.M.
Fuglsang, Katrine
Fujibe, Fumiaki
Fujii, Yasuhito
Fujimoto, Nariaki
Fukushima, Masami
Fung, Kenneth
Funkhouser, Ellen
Furgal, Christopher
Fyhri, Aslak
Gabriel, David
Gagnon, Marie P.
Galarce, Ezequiel M.
Galbraith, Shaun
Galil, Bella
Galindo-Pacheco, Gina M.
Gallagher, Bláithín Anna Mary
Gallagher, Carolyn M.
Gallardo, Antonio
Galloway, Renee L.
Galvão, Daniel
Ganapati, Emel
Gannon, Victor P.J.
Gao, Yang
Garcia, Cyril
Garcia, Valerie
García-Del-Cura, María
García-Rodríguez, José
Gardner, Andrew W.
Garnett, Stephen
Garrido, Piedad
Gasana, Janvier
Ge, Shijian
Gebbie, Kristine
Gebel, Klaus
George, Christakos
Ghosh, Rakesh
Gianella, Sara
Gianicolo, Emilio Antonio Luca
Giannuzzi, Leda
Gibb, Herman
Gibbs, Brian
Gibson, Chris L.
Giddon, Donald B.
Gilbert, Paul
Gillespie, Gordon
Gilmore, Dave
Giné Garriga, Ricard
Gioia, Rosalinda
Gissler, Mika
Glantz, Stanton
Goarant, Cyrille
Gohlke, Julia M.
Goldberg, Lynette
Goldblatt, Peter
Goldman, Howard
Goldstein, Mark K.
Golub, Elizabeth T.
Golubnitschaja, Olga
Gomez-Rubio, Virgilio
Gommel, Udo
Gonçalves, Luzia
Gong, Jianzhou
Gonzalez de Aguero, Alex
Gonzalez, Marianne Thorsen
Goodman, Annekathryn
Goodman, Melody
Goodman, Patrick
Goodrich, David
Goovaerts, Pierre
Gopinath, Bamini
Gordon, Allegra
Gordon, Catherine A.
Goris, Marga
Gowda, Nisarani K.S.
Grabenhenrich, Linus B.
Grabrucker, Andreas
Gragg, Richard
Graham, Jay
Graham, Louis F.
Grant, Alec
Graves, Alexandria
Gray, Selena
Greaves, Colin J.
Green, Heather
Greene, Scott
Greger, Manfred
Grenier, Bertrand
Grigoryan, Larissa
Grimes, Darrell Jay
Grineski, Sara
Grize, Leticia
Groen, Gunter
Grossman, Ellie
Guare, Renata
Gubbels, Jessica S.
Guerra, Marta
Guirguis, Kristen
Gulis, Gabriel
Gulrud, Kristin
Guo, Z.W.
Gushulak, Brian D.
Guski, Rainer
Ha, Eun Hee
Haagsma, Juanita A.
Haber, Steve
Habiballa, Hashim
Hackney, Madeleine
Hagen-Zanker, Alex
Haggerty, Catherine L.
Hajizadeh, Mohammad
Halder, Milimesh
Hale, Beverley
Hales, Simon
Hall, Eric
Halldorssen, Thorhallur I.
Hallstrom, Lars K.
Halonen, Jaana
Halonen, Jaana I.
Halsen, Gabriele
Halstead, Scott
Hamers, Françoise F.
Hammond, Bruce
Hammons, Amber
Hamner, Steve
Hampl, Sarah E.
Hanke, Wojtek
Hankey, C.R.
Hanna, Liz
Hanowski, Richard J.
Hansen, Alana
Hansen, Colin
Hansson, Ingrid
Harder, Marie
Hardy, Rebecca
Harkin, Kenneth R.
Harlan, Sharon
Harley, David
Harrington, Kathleen F.
Harris, Mary
Harris, Matthew
Harris, Patrick
Harris, Shelley A.
Harrison, Flo
Harris-Roxas, Ben
Hartig, Terry
Hartman, Marieke A.
Hartmann-Boyce, Jamie
Harvey, Philip
Hasan, Samiul
Hasselbalch, Steen
Hassell, Kathryn L.
Hassink, Jan
Hassiotis, Angela
Hatzopoulou, Marianne
Haub, Mark D.
Haukka, Kaisa
Hayashi, Shunji
Hayden, Mary
Hayes Jr., Don
Hayes, Bronwyn
He, Meizi
Healy, Genevieve N.
Heath, Greg
Hedberg, Craig W.
Heller, Jonthan
Henderson-Wilson, Claire
Hendryx, Michael
Hens, Niel
Hepner, Kimberly A.
Herman, Katya
Hernandez, Mark
Herr, Caroline
Hess, Jeremy
Heuch, Ivar
Heuer, Cord
Heukelbach, Jorg
Hicken, Margaret
Higgins, Niall S.
Hill, Terrence D.
Hills, Andrew P.
Hinckson, Erica
Hirvensalo, Mirja
Hiscock, Rosemary
Hitchman, Sara
Hjarnoe, Lulu
Hnatiuk, Jill
Ho, Sai Yin
Ho, Tzong-Shiann
Ho, Wen-Chao
Hoagwood, Kimberly
Hoek, Gerard
Hoenigl, Martin
Hofacre, Charles
Hofmann, Barbara
Hofstetter, Richard
Holdaway, Jennifer
Holm, Astrid Ledgaard
Holt, Kelsey
Holzel, Christina
Holzinger, Andreas
Homaira, Nusrat
Hon, Kam-lun Ellis
Hond, Elly Den
Hondula, David
Hong, Song-Iee
Hoppin, Jane
Horiuchi, Masahisa
Horn, Andrea B.
Horner, Pilar S.
Horvat, Milena
Horwitz, Pierre
Hoshiko, Sumi
Hosking, Joanne
Hothorn, Torsten
Hou, X.Y.
Houng, Harvey
House, Ron
Hout, Joseph
Howard, Natasha
Howden-Chapman, Philippa
Howell, Fenton
Hoyert, Donna L.
Hristova, Krassimira
Hsia, Jason
Hsu, Bing-Mu
Hsu, Steen J.
Hsueh, Yu-Mei
Hu, Jie
Hu, Maogui
Hu, Xuefei
Hu, Yu-Hen
Hu, Zhiyong
Huang, Gordon
Huang, Huiping
Huang, Qingxu
Huang, Yue
Huang, Zheng Y.X.
Huang, Zhuojie
Huen, Karen
Hughes, Roxanne
Huigens III, Robert W.
Hunger, Matthias
Hunt, Alistair
Hunt, Piper Reid
Hunter, Paul
Huss, Anke
Hussein, Tareq
Husslein, Peter
Hutter, Hans-Peter
Huxley, Rachel
Hwang, Bing-Fang
Hyde, Matthew
Iamarino, Mario
Ihara, Emily S.
Ikäheimo, Tiina
Ikem, Abua
Imholt, Christian
Iossifidou, Eleni
Isern, David
Isidori, M.
Jack, Darby
Jackson, Debra
Jackson, Laura
Jacobs, David R.
Jacobson, Sandra
Jahns, Lisa
Jakovljevic, Mihajlo
Jakus, Paul
James, Katherine A.
James, Peter
James, Philip
Janczukowicz, Wojciech
Jang, C.S.
Janke, Elizabeth Amy
Janssen, Sabine
Jaoude, Philippe E.
Jarnstrom, Helena
Jaume-i-Capó, Antoni
Jawad, Mohammed
Jayawardene, Wasantha P.
Jensen, Carsten
Jensen, Steen Solvang
Jeon, Byeonghwa
Jesdale, Bill
Jetter, James J.
Jia, Guang
Jimenez-Cisneros, Blanca
Jin, Li
Jit, Mark
Jjemba, Patrick K.
Johansson, AnnaKarin
Johansson, Maria
Johler, Sophia
John, Jacob
Johnsen, Svein Åge Kjøs
Johnson, Erica H.
Johnson, Evan
Johnston, Carl Gordon
Johnston, Jill
Jones, Jessica L.
Jones, Kelly
Jones, Mike
Jordan-Marsh, Maryalice
Jörg, Frederike
Jørgensen, Torben
Jorm, Louisa
Juarez, Sol Pia
Julander, Anneli
Jung, Myung-Chae
Júnior, Antônio Djalma Nunes Ferraz
Junod, Bernard
Jurasic, Marianne
Jurewicz, Joanna
Jutla, Antarpreet S.
Kabat, Geoffrey C.
Kabel, Allison
Kagawa, Masaharu
Kagawa, Takahide
Kah, Melanie
Kaiiry, Dahlia
Kaklamanou, Daphne
Kalengayi, Faustine Nkulu
Källén, Bengt
Kalokairinou, Athena
Kamada, Masamitsu
Kamm, Kelly
Kancherla, Vijaya
Kaneko, Hideo
Kannel, Prakash Raj
Kao, Chien-Ya
Karanis, Panagiotis
Karavia, Eleni
Karlsen, Randi
Käsbohrer, Annemarie
Kassa, Hailu
Katayama, Hiroyuki
Katsogiannis, Athanasios
Katz, Alan R.
Kaufman, James H.
Kaufman, James S.
Kavouras, Ilias G.
Kawai, Satoru
Kazimirova, Maria
Kearney, Greg
Keay, Lisa
Keegan, Thomas
Keene, Danya
Keleher, Helen
Kelleher, Cecily
Kempler, Peter
Kendrovski, Vladimir
Kennedy, Caitlin Elizabeth
Kern, Janet
Kerret, Dorit
Kesmodel, Ulrik Schiøler
Keun, H.C.
Key-Schwartz, Rosa
Khamisa, Natasha
Khan, Izhar
Khare, Sangeeta
Khashan, Ali S.
Khatri, Nam Raj
Kidger, Judi
Kienberger, Stefan
Kieser, Teresa M.
Kiilunen, Mirja
Kilpatrick, A. Marm
Kim, Dohyeong
Kim, Dong Soo
Kim, Heon
Kim, Hyoung-Ryoul
Kim, Ki-Hyun
Kim, Seon Tae
Kim, Sojung Claire
Kim, Woo Jin
Kim, Yangho
Kim, Young-Kee
Kimbrough, Sue
King, Brian A.
King, Mindy H.
Kingma, B.
Kioumourtzoglou, Marianthi-Anna
Kipen, Howard M.
Kirby, Joanna
Kirchengast, Sylvia
Kirk, Sara
Kirkpatrick, Sharon I.
Kishi, Reiko
Klaeboe, Ronny
Klaschka, Ursula
Kligerman, Andrew
klimes-dougan, Bonnie
Knappett, Peter S.K.
Knauper, Barbel
Knez, Jure
Knight, Theresa
Knobf, M. Tish
Knoppers, Bartha M.
Knott, Verner
Kobayashi, Takeshi
Koblin, Beryl
Koch, Manja
Koh, Wan
Kohn, Barbara
Koizumi, Nobuo
Kolehmainen, Mikko
Koletsi-Kounari, Haroula
Kollek, Daniel
Komárek, Michael
Koppe, Janna G.
Kordas, Katarzyna
Korhonen, Ilkka
Koutny, Reinhard
Kovalskyy, Valeriy
Koyama, Asuka
Kozyrskyj, Anita L.
Krajewska-Kułak, Elżbieta
Kramer, Axel
Kranz, Ashley M.
Kraut, Allen
Krawinkel, Michael B.
Krieg, Edward
Kriemler, Susi
Kroesen, Maarten
Krometis, Leigh-Anne H.
Krstic', Goran
Krug, Harald F.
Kruger, Ruan
Kryza, Maciej
Krzyzanowski, Judi
Kubota, Kengo
Kudva, Indira T.
Kujala, Urho M.
Kuligowski, Julia
Kulmala, Jenni
Kulyukin, Vladimir
Kumar, Sajeesh
Kumaragamage, Darshani
Kundi, Michael
Kuno, Rúbia
Kunst, Anton E.
Kunz, A.
Kuo, Shu-lung
Kurisu, Kiyo
Kurosawa, Kiyoshi
Kwon, Soon-Bark
Kyle, Amy
Algood, Car
L’Orange, Christian
La Rocca, Renato V.
La Torre, Giuseppe
Laas, Alo
Ladeau, Shannon L.
Ladisa, G.
Lagor, William R.
Lagzi, Istvan
Lai, Dejian
Lai, Hui-Min
Lai, W.L.
Laidlaw, Mark
Lakes, Tobia
Lal, Aparna
Lalancette, Cindy
Lalander, Cecilia
Laliberte, Nicole
Lambertini, Elisabetta
Lamm, S.H.
Lando, Harry
Laranjeira, Ronaldo
Lariccia, Francesca
Larson, Merlin D.
Larson, Timothy
Latimer, Jennifer
Lau, Colleen
Lau, Eric H.Y.
Laumbach, Robert
Laurence, Brian
Laursen, Bjarne
Lauscher, Helen Novak
Laverty, Anthony A.
Lavoué, Jérôme
Lawson, Andrew B.
Lawson, Josh
Lazzarino, Antonio
Lazzeri, Chiara
Le Bot, Barbara
Lea, Tor Erling
Lee, Brain
Lee, Duk-Hee
Lee, Gangwoong
Lee, Hian Kee
Lee, Homg-Kyu
Lee, Jae-Young
Lee, Jeong-chae
Lee, Lisa M.
Lee, Pin-Chan
Lee, Tsung Hung
Lee, Young Ji
Lefebvre, Wouter
Leisner, Jørgen J.
Leith, David
Leonard, Nola
Leong, David Tai
Leprevost, Catherine E.
Leslie, Timothy F.
Leung, Angela M.
Levallois, Patrick
Leventon, Julia
Levitas, Eliahu
Levy, Karen
Levy, Michael
Lewis, Ari S.
Lewis, Fraser I.
Li, Baoguang
Li, Chung-yi
Li, Gengbao
Li, Hong
Li, Jian
Li, Jing
Li, Jin-Hui
Li, Kaigang
Li, Lan-juan
Li, Lin
Li, Ning
Li, Peiyue
Li, Ruopu
Li, Wen-Whai
Li, Xiaofeng
Li, Xinying
Li, Xiujun
Li, Yangfan
Li, Yanli
Li, Yi-Chang
Li, Yingxia
Li, Yonghua
Li, Yu-Chuan
Li, Yuesheng
Liang, Xiao-feng
Liao, Pao-Chi
Liccardi, Gennaro
Lichtveld, Maureen
Lien, Wanyu
Lien, Yin-Ju
Lienemann, Taru
Lilja, Margareta
Lilley, Rebbecca
Lim, Chae Seung
Lim, Kyung Jae
Lin, Hsing-Juh
Lin, Kwan-Hwa
Lin, Yuan-Chung
Linder, Stephen
Lindkvist, Marie
Linos, Athena
Liou, Ming-Li
Liu, An
Liu, Chien-Tsai
Liu, Chuan
Liu, Der-Ming
Liu, Hai-Ying
Liu, He
Liu, J.C.
Liu, Jianhui
Liu, Jingling
Liu, Lei
Liu, Yongqiang
Liu, Zhongfa
Lobova, Tatiana I.
Locatelli, Clinio
Loft, Steffen
Logue, Jennifer
Loh, Miranda
Long, Maureen T.
Longmire-Avital, Buffie
Longo, Sonia
Lopez, Ana
López, T.
Loprinzi, Paul
Lovasi, Gina S.
Lovell, Geoff
Low, Chien-Tat
Lowe, Rachel
Lowenstein, Lisa M.
Lowy, Franklin Ger
Lozano-Fuentes, Saul
Lu, Jingrang
Lu, Kun
Lu, Ming-Chun
Lu, Ning
Lu, Zhiyong
Luben, Tom
Luby, Steve
Lúcio, Filipe Domingos Freires
Lugeri, Nicola
Lund, Karl Erik
Lung, Ming-Yeou
Lung, Wu-Seng
Lurie, Nicole
Lütjohann, Dieter
Luzzi, Ida
Lymberopoulos, Dimitrios K.
Lynne, Dawkins
MacCalman, Laura
MacCarthy, Sarah
Macfarlane, Duncan
MacIntyre, Elaina A.
Mackowiak, Philip A.
Macleod, Catriona
Macmillan, Alex
Macy, Jonathan T.
Madronich, Sasha
Magliano, Dianna J.
Mai, Weiyi
Malecki, Kristen
Målqvist, Mats
Manby, Martin
Manca, Stefania
Mancini, Laura
Manco, Altay
Mandel, Jeff
Mangal, Tara
Mann, Koren K.
Mannello, Ferdinando
Mannino, David M.
Marc, Daniel
Margolis, Helene
Márialigeti, Károly
Marian, Mary
Marin, Daniela E.
Mariottini, Gian Luigi
Maritz, Gert S.
Markevych, Iana
Marreiros, Goretti
Marsche, Gunther
Marsh, Gary
Martin, Andrew
Martin, Karen
Martinasek, Mary
Martinez Dominguez, Francisco
Martínez Fernández, Paloma
Martin-Olmedo, Piedad
Martins, Fernando Gomes
Martins, Maria O.
Masala, Giovanna
Maschke, Christian
Mateus, Marcos
Mathes, Robert
Mathur, Manu
Matschke, Jakob
Matthews, Charles E.
Mattoo, Autar
Mattson, Mats-Olof
Maunula, Leena
Mayer-Scholl, Anne
Mayer-Wagner, Susanne
Mcaughey, John
McBride, Graham B.
McCabe, Sean Esteban
McCarty, Carolyn
McCormick, Sabrina
McCrone, Paul
McCurdy, Karen
McDiarmid, Melissa A.
McDonald, Katherine E.
McGuire, Sarah
Mckay, Michael T.
Mckee, Georgina
McKelvey, Karma
McKenzie, Lisa
McLaren, Lindsay
McLaws, MaryLouise
McLay, James
McLean, Susannah
McManus, Hamish
McNaughton, David
McRobbie, Hayden
McSpirit, Stephanie
Medek, Danielle
Meisinger, Christa
Melike, Jaymie
Melintescu, A.
Menai, M. Medhi
Menne, Bettina
Merget, Rolf
Meschke, Scott
Messi, Patrizia
Mezuk, Briana
Micciolo, Rocco
Siu, Kin Wai Michael
Michelozzi, Paola
Michels, Alexander J.
Michimi, Akihiko
Mielke, Howard
Mikhailova, Elena
Milic, Sonja
Millán Calenti, José C.
Miller, Mark
Milte, Catherine
Miltner, Anja
Min, Kyung Rok
Minaker, Leia
Mingomataj, E.C.
Mínguez-Alarcón, Lidia
Minguillón, María Cruz
Minnes, Sonia
Minutolo, Filippo
Mirabelli, Maria C.
Mirauda, Domenica
Mirza, M. Monirul Q.
Misra, Ranjita
Mitas, Josef
Mitrakas, Manassis M.
Mitrakou, Asimina
Mitrani-Reiser, Judith
Mitsakou, Christina
Mo, Jinhan
Moglia, Magnus
Moholdt, Trine T.
Mokrech, Mustafa
Moliner-Urdiales, Diego
Molinos-Senante, María
Møller, Henrik
Mölter, Anna
Moncharmont, Bruno
Monserrat, Maria Ramos
Monte, Luis
Montesano, Lius
Monyeki, Andries
Moodie, Crawford
Moodle, Indres
Mooijaart, Simon P.
Moore, George
Moore, L.E.
Moore, Roland
Morabito, Marco
Moreno, Jennette Palcic
Moretto, Angelo
Morgan, Lloyd
Morin, Cory
Morland, Kimberly
Morojele, Neo
Morris, Viola B.
Morrissey, Anne
Morrone, Michele
Mortensen, Mary E.
Mortimer, Kevin
Mortimer, Michael J.
Mosler, Hans-Joachim
Mota, Jorge
Moura, L.
Mrozek-Budzyn, Dorota
Mufson, Laura
Muggeo, Vito M.R.
Mukherjea, Arnab
Mulè, Giuseppe
Mullan, Judy
Müller, Bettina
Muller, Marc
Muller, Matthew D.
Mundi, Manpreet S.
Munoz, Flor M.
Muñoz-Zanzi, Claudia
Munro, Neil
Murakami, Keiko
Murakami, Michio
Murante, Anna Maria
Murbach, Manuel
Murdoch, David R.
Murphy, Susan Kay
Musiat, Peter
Mutowo, Mutsa
Mytum, Harold
Naffakh, Nadia
Nagai, Haruyasu
Nagel, Susan C.
Naidoo, Saloshni
Naidu, Rahul
Naik, Reshma
Naleway, Allison
Nalls, Mike
Nardell, Edward
Nargis, Nigar
Narita, Daiju
Narvaez, Claudia
Khalili, Nasrin R.
Nastos, Panagiotis
Natale, Vincenzo
Nazare, Barbara
Nazare, Julie-Anne
Ndione, Jacques-André
Nebot, Carolina
Nederhof, Esther
Negri-Cesi, Paola
Nelson, Erik J.
Nena, Evangelia
Netzband, Maik
Neumann, Tim
Neupane, Subas
Névéol, Aurélie
Newton, Robert
Ng, Chris Fook Sheng
Nicaise, Pablo
Nicholls, Stuart G.
Nickel, Nathan
Nidzgorska-Lencewicz, Jadwiga
Nielsen, Jens
Nielsen, Morten
Nienhaus, Albert
Nikolov, Jovana
Nilsen, Tom Ivar Lund
Nin, Jordi
Niyogi, Dev
Nord, Carl Erik
Norström, Madelaine
Noti, John
Nowicki, Michal
Núñez Torres, María Isabel
Nutt, David
Nyangweso, Mary
Oakes, Michelle
O’Brien, Liz
O’Connor, Teresia
Odland, Jon Øyvind
Odoi, Agricola
Oehmen, Adrian
Ogawa, Wakano
Ogden, Nicholas H.
O’Hara, Marilyn
Ohhashi, Akiyoshi
Oiamo, Tor Henning
Ojala, Ann
Okechukwu, Cassandra
Oksa, Juha
Okwo-Bele, Jean-Marie
Oliveira, Ana
Oliveira, M. Beatriz P.P.
Omoruyi, Felix
Omumbo, Judy
Ong, Mei-Sing
Onodera, Takashi
O'Reilly, Ciara E.
Organelli, Emmanuelle
Orru, Hans
O'Sullivan, Tracey L.
Over, Eelco A.B.
Owens, Janine
Oxley, Tim
Pääkkönen, Rauno
Pachucki, Mark C.
Padula, Amy M.
Pagès, Frédéric
Pagulayan, Kathleen
Pai, Tzu-yi
Paju, Susanna
Palacios-Ceña, Domingo
Palamara, Anna
Pallan, Miranda J.
Pallesen, Ståle
Pallier, Virginie
Palm, Noah Wolcott
Palm, Ulo
Pan, Erica
Panis, Luc Int
Pantelopoulos, Alexandros
Panuwet, Parinya
Papadopoulos, Nikos
Papp, András
Pardies, Yin
Pardo, Tania
Pareeth, Sajid
Parekh, Sanjoti
Park, Jae Bum
Park, Jung-duck
Park, Robert
Parks, Christine
Parnell, Winsome
Parparov, Arkadi
Parrott, Wayne
Partonen, Timo
Partridge, Bradley
Paschall, Mallie
Paterson, Beverley
Patole, Sanjay
Patrick, Bob
Pattaro, Cristian
Patterson, Freda
Patton, Allison
Paulson, Nels
Pauly, John L.
Paunović, Katarina
Payne, Thomas J.
Payne-Sturges, Devon
Pazdiora, Petr
Pearce, Dora Claire
Pearce, Elizabeth N.
Pearce, Mark
Pearson, Jennifer L.
Pedersen, Charlotte Gjørup
Pedersen, Marie
Peletto, Simone
Pelkey, Neil
Pelletier, Guillaume
Peltzer, Karl
Peña-Fernández, A.
Peng, Chiung-Yu
Penn, Linda
Pennig, Sibylle
Pentecost, Allan
Pepper, Jessica K.
Pereira, Dolores
Pereira, Joana L.
Pereira, Martha
Peretz, Avi
Perez, Rose
Perin, Jamie
Perkins, Kenneth
Perrucci, S.
Persoz, Charles
Pesch, Beate
Peter, Richard
Peters, J.L.
Petersen, Eskild
Petkova, Elisaveta Petkova
Petrić, Dušan
Petricevic, Ljubomir
Petrie, Dennis
Pfleiderer, Sebastian
Phillips, Rhonda G.
Piatt, Gretchen A.
Pica, Natalie
Picardeau, Mathieu
Pichini, Simona
pickering, Amy
Pickles, Michael
Pina-Watson, Brandy
Pinsky, Benjamin A.
Pinti, Marcello
Pinto, Bernardine
Pintó, Rosa M.
Pira, Enrico
Pirani, Elena
Pirozzi, Cheryl S.
Pisani, R.
Piskorska-Pliszczynska, Jadwiga
Pistelli, Francesco
Pitt, Henry A.
Plant, Roel
Pleil, Joachim Dieter
Pohanka, Miroslav
Poirier, Miriam
Polgreen, Philip
Pollitt, Rodney J.
Polosa, Riccardo
Polyak, Steven
Polychronopoulou, Argy
Pompili, Maurizio
Ponnet, Koen
Pope III, C. Arden
Porru, Stefano
Porteous, Nuala
Posnack, Nikki Gillum
Possel, Patrick
Pottee, Kevin
Poucheret, Patrick
Poulymenopoulou, Mikaela
Pragst, Fritz
Prati, Gabriele
Pratt, Angela
Pretorius, Richard W.
Preve, Nikolaos
Prevots, Rebecca
Price, Bronwyn
Price, Matt A.
Prochaska, John
Procopio, Jesús R.
Proctor, Deborah M.
Prosper, Justin
Pruden, Amy
Pu, Christy
Puddick, Jonathan
Pulugurtha, Srinivas S.
Punch, Jerry L.
Pyrzynska, Krystyna
Raffa, Jesse
Raghupathi, Wullianallur
Rahman, Bayzidur
Rahman, Khaja Zillur
Raina, Komal
Raina, Parminder
Rainisch, Bethany
Räisänen, Sari
Raitakari, Suvi
Rajagopalan, Neethi
Rajagopalan, Priya
Rajamani, Sripriya
Ralevski, Elizabeth
Ramasamy, Ranjan
Ramirez, Clemencia
Rand-Hendriksen, Kim
Rappuoli, Rino
Rasmussen, Jon B.
Raso, Giovanna
Ravnskov, Ulf
Razzouk, Denise
Ready, Paul D.
Rebolj, Matejka
Rechel, Bernd
Recio-Rodriguez, Jose I.
Reefhuis, Jennita
Reeves, Aaron
Regmi, Pusker
Reichert, Monika
Reid, Gregor
Reid, Robert J.
Reimers, Anne
Reisen, William K.
Rem, Peter
Remoundaki, E.
Remuzzi, Giuseppe
Ren, Yuan
Rene, Eldon R.
Renzaho, Andre
Rey, Grégoire
Rhodes, Scott Duane
Riccio, Angelo
Rice, Glenn E.
Rich, Jane
Richards, Russell
Richmond, Janet
Richmond-Bryant, Jennifer
Ridefelt, Peter
Rider, Cynthia
Ridgers, Nicky
Riedel, Natalie
Riediker, Michael
Rietra, Rene
Rim, Donghyun
Riordan, James
Risher, John F.
Risley-Curtiss, Christina
Risser, Ralf
Risso, Davide
Robbins, Cheryl L.
Roberson, Donald N.
Roberts, Adam
Roberts, Michael W.
Robertson, Colin
Robertson, L.J.
Robinson, Jude
Robledo, Candace
Robson, Mark
Rocheleau, Carissa
Rocklöv, Åsa Holmner
Rodenburg, Lisa
Rodrigues, António S.
Rodrigues, Irene
Rodriguez, Jose
Rodriguez, Marcela
Rodríguez-Martín, Jose Antonio
Roeder, Stefan
Rogan, Eleanor
Rohr, Linda E.
Romagna, Giorgio
Romalde, Jesús López
Romero, Eliete Caló
Romero, Gustavo A.S.
Rooks, Ronica N.
Rosário, Rafaela
Rosenkranz, Richard R.
Ross, Joshua
Rossi, François
Rossignol, Dan
Rowa-Dewar, Neneh
Rowe, Michael
Rowson, Steven
Royer, Isabelle
Rubin, James
Rubinelli, Sara
Rubio, Doris McGartland
Rucci, Paola
Rue, Montserrat
Rugg, Julie
Rugh, Douglas
Rugkasa, Jorun
Ruiz-Ruiz, Carmen
Rundle, Andrew
Rüsch, Nicolas
Ruud, Torleif
Ryadnov, Max
Ryan, Michael
Rygaard, Martin
Sabatini, Angelo
Sackett, Dana K.
Sadler, Richard
Sæbø, Gunnar
Sægrov, Sveinung
Saez, Marc
Sagatun, Åse
Sagiv, Sharon K.
Saint-Amour, Dave
Saito, Mitsumasa
Sakan, Sanja M.
Salam, Mohamed Abdel
Salinas, Carla
Salzer, Jonatan
Samadi, Sadegh
Sammito, Stefan
Samnaliev, Mihail
Sánchez, José Luis Reyes
Sánchez, María
Sanchez-Zapata, Elena
Sancini, Giulio
Sandanger, Torkjel M.
Sanders, Diethard
Sandifer, Paul
Sanny, Charles G.
Sano, Daisuke
Santaella, Catherine
Santos, Ana Paula Pires Dos
Santos, J.L.
Sarfaty, Mona
Sargent, L.M.
Sarin, Enisha
Satariano, William
Sato, Yukita
Satoh, Masahiko
Sauer, Pieter
Saul, Nadine
Sauleau, Erik-André
Sauni, Riitta
Sawa, Tomohiro
Schaffer, Andrea
Schauss, Alexander G.
Schenk, Linda
Schenker, Marc
Schijven, Jack F.
Schikowski, Tamara
Schimmenti, Adriano
Schmitt, Carol
Schneider, Arthur B.
Schneider, Raphaël
Schoeler, Natasha E.
Schoen, Cathy
Schreckenberg, Dirk
Schreinemachers, Dina
Schuller, Hildegard M.
Schulte, P.A.
Schulz, Peter J.
Schumann, Barbara
Schuna, John
Schuster, Michael
Schütte, Martin
Schützwohl, Matthias
Schuurman, Nadine
Schwaiger, Karin
Scobie, Linda
Scognamiglio, Viviana
Sebastiani, Paola
Seeley, Janet
Sella, Luca
Selling, Katarina Ekholm
Sembajwe, Grace
Semple, Sean
Seong, J.C.
Serrano, Antoni
Sexton, Julie M.
Sexton, Ken
Shaddick, Gavin
Shama, Gilbert
Shanks, Leslie
Shannahan, Jonathan
Shao, Kwang-Tsao
Shao, Yongzhao
Sharkey, Amanda
Sheffield, Jeanie
Sheffield, Jeanne K.
Shefi, Orit
Sheinkopf, Stephen
Shepherd, Daniel
Sherar, Lauren
Sheriff, Glenn
Shi, Yuyan
Shi, Zhenqing
Shields, Jennifer
Shima, Masayuki
Shin, Jung-hye
Shin, Won Sop
Shochet, Ian
Shoenfeld, Yehuda
Shrader-Frechette, Kristin
Shriar, Avrum J.
Siceloff, E. Rebekah
Sicotte, Diane
Sidebotham, Peter
Siegel, Robert
Sieminska, Alicja
Sigmund, Erik
Silva, Edson Luiz
Silva, Vandermi
Silva-Sanigorski, Andrea
Silventoinen, Karri
Simal-Gándara, Jesús
Simeonov, V.
Simon, O.
Sinclair, Ryan
Singh, Vikas
Sironen, Tarja
Sisson, James C.
Sit, Cindy Hui-Ping
Sixsmith, Judith
Sjögren, Tuulikki
Skalska, Kinga
Skiadas, Christos H.
Skomorowski, Marek
Skön, Jukka-Pekka
Skouloudis, Andreas
Skousen, Jeffrey
Skrha, Jan
Slater, Matt
Slater, Sandra J.
Sloan, Chantel
Smani, Younes
Smieja, Marek
Smith, Allan
Smith, Andy
Smith, George Davey
Smith, James
Smith, Lee
Smith, Len
Smith, Neil
So, Chi-Chiu
Soares, Felix
Søgaardc, Karen
Sokolova, Ekaterina
Solcan, Carmen
Solimini, Renata
Son, Nguyen-Thanh
Sone, Hideko
Soneji, Samir
Song, Xiaobo
Soo, Jhy-Charm
Sørensen, Jacob Thorstedxc
Sørensen, Mette
Sospedra, Javier
Souissi, Sami
Sousa, Mário
Spanier, Britta
Spear, Robert C.
Spector, Logan G.
Spence, Nicholas
Spence, Sue
Spencer-Hwang, Rhonda
Sperling, Michael
Spickett, Jeffery
Sreekanth, Janardhanan
Ssematimba, Amos
St.Helen, Gideon
Stabile, L.
Stack, Steven
Stagnitti, Karen
Stallings, Jonathan D.
Stam, Rianne
Stamatis, Nikolaos
Standl, Eberhard
Stanhewicz, Anna E.
Stansbury, Kim
Starcke, Katrin
Staten, Lisa
Stead, Lindsay F.
Stefanidou, Maria E.
Stein, Leah
Steinberg, Michael B.
Steinhauser, Georg
Stelzer, Jiri
Stewart, Iain
Stewart, Jill
Stieb, David M.
Stoecker, Charles
Stoklosa, Michal
Story, Michael
Straka, Vera
Stratton, Gareth
Strobino, Donna
Strohmeier, Dagmar
Stronks, Karien
Strugnell, Claudia
Styblo, Miroslav
Su, Chang
Su, Chunming
Su, Shew-Jiuan B.
Suárez-Estrella, F.
Suarez-Varela, Maria Morales
Subramaniam, Mythily
Sucharew, Heidi
Sugiura, Shinichi
Suikkanen, Sanna
Suliburska, Joanna
Sumi, Ayako
Summers, Huw
Sumner, Walton Ii
Sun, Danfeng
Sun, Hongmin
Sun, Tao
Sun, Tonghua
Sun, Wenjie
Sung, Valerie
Suputtamongkol, Yupin
Sureda, Antoni
Sussman, Steven Y.
Suter, Melissa A.
Suwazono, Y.
Suzuki, Kohta
Svoboda, Zdenek
Sze, Daniel
Szymańska, Jolanta
Szyszkowicz, Mieczysław
Tabak, Izabela
Tainio, Marko
Takahashi, Masaya
Takeuchi, Masaru
Talbot, Prue
Talbott, Evelyn O.
Talhout, Reinskje
Tally, Steven
Tamashiro, Hiko
Tamí-Maury, Irene
Tan, Jianguo
Tang, Zhaoxin
Tartari, Gianni
Taweel, Adel
Taxvig, Camilla
Taylor, Caroline
Taylor, Kathryn
Taylor, Myra
Taylor, Natalie J.
Taylor, Nicholas F.
Taylor, Sally
Tchicaya, Anastase
Tellez, Guillermo
Templeton, Michael
Tenerelli, Patrizia
Teng, Tjoon Tow
Teo, Jeanette W.P.
Teodoro, Cordova-Fraga
Testa, Maria
Thatcher, Andrew
Thompson, Lisa
Thompson, Marcella Remer
Thomson, Neil C.
Thornbush, Mary
Thorne, Robert
Thornes, John E.
Thornton, Lukar
Thow, Anne Marie
Tijing, Leonard
Tinanoff, Norman
Tisminetzky, Mayra S.
Titze, Sylvia
Tobias, Jim
Tobin, Karin E.
Töero, Klára
Tol, Richard
Toloo, Sam
Tomatis, Maura
Tomei, Gianfranco
Tomkinson, Grant
Tomoda, Fumihiro
Tonetti, Lorenzo
Tong, Haiyan
Torrey, William C.
Tortosa Edo, Vicent
Toscano, William A.
Tosini, Fabio
Toth, John D.
Townshend, Tim
Toyokuni, Shinya
Trail, Frances
Tremblay, Mark
Triantafyllidou, Simoni
Trinchieri, Giorgio
Tsai, Kun-Hsien
Tsai, Luke Y.
Tsai, Perng-Jy
Tsai, Yuh-Show
Tsang, Sandro
Tsezos, Marios
Tsow, Francis
Tu, Jun
Tudor, T.L.
Tumwebaze, Innocent K.
Tung, C.J.
Turner, Benjamin F.
Turner, Samantha
Turvey, Carolyn
Tutenges, Sebastien
Tyson, Julian
Uchiyama, Shigehisa
Udias, Angel
Unfried, Klaus
Unicomb, Anne
Vaccari, Francesco Primo
Vainieri, Milena
Val Del Río, Angeles
Valavanidis, Athanasios
Valent, Francesca
Vallero, Daniel
Van Audenhove, Chantal
Van Baak, Marleen A.
Van Bogaert, Peter
Van Bruggen, Ariena
Van Cauwenberg, Jelle
van den Berg, Agnes
Van Den Branden, Sigrid
van den Kommer, Tessa N.
van Dijk, M. Pieter
Van Hoorenbeeck, Kim
Van Sluijs, Esther
van Voorhees, Benjamin
van Zyl, Hendra
Vanclay, Frank
Vandenberg, Laura
Vander Stichele, Robert
VanDerslice, Jim
Vanneste, Sven
Vargo, Jason
Vassura, Ivano
Vaughan, Ashley M.
Vayenas, Dimitris
Vaz Moreira, Ivone
Vazquez Benitez, Gabriela
Vella-Zarb, Rachel
Venkatesh, Mohan Pammi
Ventikos, Nikolaos P.
Verheij, Robert A.
Verhoeff, Arnoud
Verhoughstraete, Marc
Verlato, Giuseppe
Verloigne, Maïté
Verschaeve, Luc
Verstraete, Alain
Verykouki, Eleni
Viani, Rolando M.
Vias, J.M.
Viazzi, Francesca
Vieira, Edgar Ramos
Vienneau, Danielle
Vignali, Claudia
Vila-Corcoles, Angel
Vilavert, Lolita
Villagra, Paula
Villalbi, Juan Ramon
Villanueva, Karen
Vinik, Aaron
Vinikoor-Imler, Lisa C.
Viscogliosi, Giovanni
Viscusi, W. Kip
Vitali, Matteo
Vohra, Salim
Vollstädt-Klein, Sabine
Volpe, Robert
von Esenwein, Silke
von Stumm, Sophie
Voss, Jameson D.
Wada, Mitsuhiro
Wade, Eric R.
Wade, Rebecca
Wade, Tim
Wagener, Theodore L.
Wagner, Sara
Wahlbeck, Kristian
Walczak-Jędrzejowska, Renata
Waliszewski, Stefan M.
Walker, Edward D.
Walker, J.T.
Walkowiak, Wladyslaw
Walla, Peter
Wallace, J.M.
Wallmann-Sperlich, Birgit
Walton, AnnMarie
Walz, Riger
Wanderley-Teixeira, Valeria
Wang, Fahui
Wang, Haiyan
Wang, He
Wang, Hong
Wang, Hsiang-Chen
Wang, Jiechen
Wang, May C.
Wang, Pi-Chung
Wang, Shaobin
Wang, Shengrui
Wang, Shoulin
Wang, Shu-Li
Wang, Szu-Hua
Wang, Xingfeng
Wang, Yayi
WANG, Yi
Wang, Yuanpeng
Wang, Zhiwei
Wang, Zhong Q.
Wang, Zijian
Ward, Mary
Ward, Wendy
Wardrop, Nicola
Ware, Lisa
Waring, Rosemary
Warnakulasuriya, Saman
Warshawsky, Daniel Novik
Watanabe, Toshihiro
Waterlot, Christophe
Waters, Clarice N.
Watt, Susan
Waxman, Alan G.
Webb, Christian
Webster, Jacqui
Wegener, Sandra
Wei, Bao
Weinmayr, Gudrun
Weinstein, Sally
Welch, David
Weller, Michael G.
Wells, Nancy
Wen, Tzai-Hung
Wennerstrom, Ashley
Werneburg, Brooke L.
Westbrook, Cherie
Westerhoff, Paul
Westerterp, Klaas R.
Weyandt, Lisa
Wheeler, David C.
White, Richard
White-Newsome, Jalonne
Wickliffe, Jeffrey
Widerström, Micael
Wieczorowska-Tobis, Katarzyna
Wiegant, Fac
Wiggers, John
Wilke, Olaf
Williams, Alison L.
Williams, D'Ann L.
Williams, David M.
Williams, Mary
Williams, Paul
Williams, Philip
Williams, Susan
Willox, Ashlee Cunsolo
Wilson, A. Peter R.
Wilson, Anne
Wilson, Leigh
Wilson, Peter
Windsor, L. Jack
Winkler, Inga
Winkler, Mirko
Wint, William
Winters, Carena
Winters, Meghan
Wise, Matt P.
Wittcopp, Chrystal
Woith, Wendy
Wolmer, Leo
Wong, Bryan
Wong, Chit-ming
Wong, Emmy M.Y.
Wong, Hai Ming
Wood, Andrew W.
Wood, W. Gibson
Woodfield, Lorayne
Woodman, Nick
Woolhouse, Mark
Wordlaw-Stinson, LaShawn
Worsley, Anthony
Wright, Caradee
Wright, Jim
Wright, Mark Mba
Wright, Rosalind J.
Wu, Aibo
Wu, Jun
Wu, Trong-neng
Wu, Yuzhe
Wunder, Elsio
Wyszkowska, Jadwiga
Xiao, Liwen
Xie, Shuguang
Xierali, Imam M.
Xu, Fenglian
Xu, Howard
Xu, Xiaobin
Xu, Xiaohui
Xu, Zhengping
Yang, Albert C.
Yang, Rong Sen
Yang, Yizhao
Yao, Gang
Yap, Marie
Yassi, Annalee
Yeatts, Karin
Yeh, Kuo-Wei
Yengoh, Genesis
Yim, Un Hyuk
Yin, Zenong
Yiu, Cynthia K.Y.
Yokoyama, Yoshihito
Yoo, Eun-Hye
Yoon, Hyeun Joong
Yoon, Tae Ki
Yoshimasu, Kouichi
Yoshinaga, Jun
Yoshinaga, Shinji
Young, Jami
Youngstedt, Shawn
Yu, Jae-Hyuk
Yu, Liang-Chih
Yu, Tsung-Hsien
Yu, Xuejie
Yuan, Dongxing
Yun, Katherine
Zabiegała, Bożena
Zacchini, Massimo
Zahran, Hatice S.
Zaichkowsky, Leonard
Zandman-Goddard, Gisele
Zaragoza, Guillermo
Zeeb, Hajo
Zeliadt, Steven
Zellmer, Sebastian
Zeman, Petr
Zeng, Guang Ming
Zhang, Bing
Zhang, Jian
Zhang, Jinliang
Zhang, Lei
Zhang, Qingpeng
Zhang, Wanqi
Zhang, Xun
Zhang, Yan
Zhang, Zhengdong
Zhao, Hongtao
Zhao, Huazhang
Zhao, Jinshun
Zhao, Tingting
Zhao, Yun
Zheng, Xinqi
Zhou, Changlin
Zhou, Yibiao
Zhu, Dauw-Song
Zhu, Motao
Zhu, Qian
Zota, Ami
Zou, Jin
Zougagh, Mohammed
Zuccato, Ettore
Zuniga, Genny Carrillo
Zurek, Ludek
Zvoleff, Alex

